# Quercetin reverses docetaxel resistance in prostate cancer via androgen receptor and PI3K/Akt signaling pathways

**DOI:** 10.7150/ijbs.41686

**Published:** 2020-02-10

**Authors:** Xinxing Lu, Feiya Yang, Dexi Chen, Qinxin Zhao, Dong Chen, Hao Ping, Nianzeng Xing

**Affiliations:** 1 Department of Urology, Beijing Chaoyang Hospital, Capital Medical University, Beijing, P.R. China; 2 Department of Urology, National Cancer Center/Chinese Academy of Medical Sciences Cancer Institute and Hospital, Chinese Academy of Medical Sciences and Peking Union Medical College, Beijing, P.R. China; 3 Beijing You'an Hospital, Capital Medical University, Beijing, P.R. China; 4 Department of Urology, Beijing Tongren Hospital, Capital Medical University, Beijing, China

**Keywords:** prostate cancer, docetaxel, resistance, quercetin, androgen, PI3K, Akt, stem cell, EMT, P-gp

## Abstract

Docetaxel is the first-line chemotherapy agent for metastatic prostate cancer. However, the emergence of resistance diminishes its efficacy and limits the survival benefit. Quercetin is a dietary flavonoid which has been shown to have multiple anti-cancer effects. Also, quercetin has been reported to reverse chemo-resistance in many other cancers. This study was to determine whether quercetin could reverse docetaxel resistance in prostate cancer cells and xenograft models, thereby exploring the underlying mechanism. Depending on the docetaxel-resistant cells (LNCaP/R, PC-3/R) which were established from docetaxel-sensitive cells (LNCaP, PC-3), it was demonstrated that quercetin could reverse docetaxel resistance in prostate cancer on proliferation, colony formation, migration, invasion and apoptosis. Although single docetaxel application had little effect on docetaxel-resistant cells, combining docetaxel with quercetin was significantly effective. Combination therapy could maximally inhibited PI3K/Akt pathway and promoted apoptosis. As shown by *in-vivo* study, xenograft tumors treated by docetaxel with quercetin had poorest growth. Then, to investigate the underlying mechanisms, the differences among parental cells, docetaxel-resistant subclones and quercetin treated resistant subclones were evaluated. It was found that docetaxel-resistant subclones had stronger activation of androgen receptor and PI3K/Akt pathway, more remarkable mesenchymal and stem-like cell phenotypes, and more P-gp expression than that of parental cells. Interestingly, quercetin could reverse these transformations. Our data revealed that quercetin had docetaxel-resistance reversal effect both *in vitro* and *in vivo* and provided in-depth support for clinical use of quercetin in docetaxel-resistant prostate cancer.

## Introduction

Prostate cancer is the most common malignancy and the second leading cause of cancer death for males in the US with estimated 174,650 new cases and 31620 deaths within US during 2019 1. Many cases of prostate cancer are detected in metastatic stages, or progress to metastatic stage after the reception of initial treatment 2. Docetaxel (Doc) is the first-line treatment against metastatic prostate cancer 3. Docetaxel is an anti-mitotic agent, which is able to restrain microtubule disassembly and has been identified to down-regulate androgen receptor (AR) transcription. Besides, it can also impede the translocation of AR to nucleus in response to both androgens and ligand-dependent signaling pathways 4, 5. Docetaxel also decreases the expression of AR by binding to gene promoter and up-regulates Forkhead box O1 (FOXO1), which is a strong transcriptional repressor of AR 6. However, it has some limitations, for example, either acquired or intrinsic drug resistance which is a major cause for the failure of therapy. Although several reversal agents of docetaxel resistance have been found, few of them can be used in current clinical settings due to the poor reversal effect or severe side effects 7, 8. Therefore, there is an urgent need to probe into the mechanisms of docetaxel resistance and find a novel way to overcome these obstacles faced by urologists.

Quercetin (3, 3', 4', 5, 7-pentahydroxyflavone, Quer), is a bioactive flavonoid which is widely distributed in vegetables and fruits, such as green tea, onions, apples, and red wine. It has been reported with many beneficial properties including antioxidant, anti-inflammatory, and anti-cancer activities both *in vitro* and *in vivo* 9-11. Our previous study also showed that quercetin could suppress human prostate cancer cell and human prostate cancer cell xenograft growth through multiple signaling pathways. In addition, unlike the conventional chemotherapy, it could be harmless to normal cells 12-14. Moreover, accumulating evidence indicated that quercetin could sensitize multidrug-resistant cell lines to some chemotherapeutic agents 14. However, whether quercetin could reverse docetaxel-resistance had not been verified yet.

Since the publication of the results from randomized controlled trials STAMPEDE 15 and CHAARTED 16, docetaxel had become the first-line treatment for metastatic castration resistant prostate cancer (mCRPC) as well as metastatic hormone sensitive prostate cancer (mHSPC). Although there were studies about combination therapy of green tea and quercetin which sensitized PC-3 cells and xenograft to docetaxel chemotherapy 17, 18, few studies had focused on the combination of docetaxel and quercetin therapy for HSPC treatment.

In the present study, docetaxel-resistant prostate cancer cell lines were established from hormone sensitive prostate cancer (HSPC) LNCaP and castration resistant prostate cancer (CRPC) PC-3 to mimic clinical docetaxel resistance. For the first time, the results verified that quercetin had docetaxel resistance reversal effect in prostate cancer both *in vitro* and *in vivo*. In addition, this was the first study which evaluate the potential to combine docetaxel and quercetin for the treatment of HSPC, and depending on the results, the combination therapy showed advanced efficacy to comply with the treatment concept at present.

## Materials and Methods

### Chemicals and reagents

Docetaxel was purchased from Selleck Chemicals (Houston, USA); Quercetin and dimethyl sulfoxide (DMSO) were purchased from Sigma (St. Louis, MO, USA).

### Cell Lines, cell culture and docetaxel-resistance induction

Human prostate cancer LNCaP (HSPC) and PC-3 (CRPC) cell lines were obtained from Peking Union Medical College. These two kinds of prostate cells were cultivated in RPMI-1640 medium (Hyclone, Logan, UT) supplemented with 10% fetal bovine serum (Hyclone, Logan, UT) and maintained in an incubator containing 5% CO^2^ at 37°C. Docetaxel-resistant prostate cancer cells (LNCaP/R, PC3/R) were established by continuous exposure to increasing concentrations of docetaxel. As there was no standard method for the docetaxel resistance induction, a unique method was established to develop docetaxel-resistant cells. In brief, all kinds of prostate cancer cells were incubated with RPMI-1640 medium supplemented with 10% fetal bovine serum under exposure to the docetaxel doses of 0.1 nM, 0.2 nM, 0.5 nM, 1 nM, 5 nM or 10 nM in sequence. The dose of docetaxel was increased step by step, and the exposure to the doses was supposed to last for about 30 days. In the course of induction, especially before elevating the concentration of docetaxel, the conditions of cells were under tight monitoring. If many dead cells appeared or many cells cloned very slowly, it should be delayed to elevate the concentration of docetaxel. Once cells were freely dividing in the medium with 10 nm docetaxel, they were considered docetaxel-resistant and labeled as LNCaP/R or PC3/R. Then, the docetaxel-resistant cell lines were used for the subsequent sections.

### Cell proliferation assay (CCK-8 Assay)

CCK-8 kit (KeyGEN BioTECH, China) was applied to investigate cell proliferation. The cells in the logarithmic growth phase were seeded into 96-well plates at the density of 5×10^3^ cells per 100 µL and cultured at 37˚C and 5% CO^2^ in triplicate. After cultured for 24h to enable the attachment of cells, cells were treated for further 24h with serial concentrations of quercetin (Quer/µM: 5, 10, 20, 50, 100, 150, 200), docetaxel (Doc/nM: 1, 5, 10, 20, 50, 100) , vehicle control (DMSO) or blank control. Two hours before the termination of each time points, 10 µl CCK-8 was added to each well and co-cultured with cells for another 2h in humidified environment containing 5% CO^2^ at 37˚C. The optical density (OD) of each well was measured with a microplate reader (Bio-Rad Laboratories, Inc., Berkeley, CA, USA) at the wavelength of 450 nm. The inhibition curves of quercetin and docetaxel for each kind of cells were achieved. And then, the concentration which caused about 20%-60% cell growth inhibition for chemo-sensitive cells (LNCaP and PC-3) was selected for the subsequent combination study. These cells were treated with the following reagents for 24h or 48h: blank control, vehicle control (DMSO), 20 μM Quer, 5 nM Doc, 20 nM Doc or 20 μM Quer + 5 nM Doc. Two hours before the termination of each time points, 10 µl CCK-8 was added to each well and co-cultured with cells for another 2h in humidified environment containing 5% CO^2^ at 37˚C. The optical density (OD) of each well was measured with a microplate reader (Bio-Rad Laboratories, Inc., Berkeley, CA, USA) at the wavelength of 450 nm.

### Colony formation assay

LNCaP/R and PC-3/R cells were treated for 48h with vehicle control, 5 nM Doc, 20 μM Quer or 5 nM Doc + 20 μM Quer. Then cells were trypsinized and dispensed into individual wells of 6-well culture dishes with the density of 200 cells per well, followed by incubation for 10 days without changing the medium. 10% formaldehyde was used to fix cell colonies for 10 minutes, and 0.5% crystal violet was employed to stain cell colonies for 5 minutes. After that, the number of colonies containing> 50 cells was counted under a dissecting microscope. The percentage of cell survival was calculated. The experiment was performed in triplicate.

### Cell migration assay (wound healing assay)

Cell migration capacity was measured by wound healing assay. LNCaP/R and PC-3/R cells were transplanted in 6-well plates. Afterwards, A sterile 200-μl pipette tip was used to scratch a vertical line in the cell plate until 90% confluency was achieved. Cell debris were washed off with PBS twice. Then cells were treated for 48h with serum-free 1640 containing any one of the following ingredients: vehicle control (DMSO), 10 μM Quer, 5 nM Doc, 10 μM Quer + 5 nM Doc. Images were obtained in triplicate for each condition with inverted microscope at 0h to acquire baseline images, and at 48 h which was the end-point of the assay. Wound-healing area was calculated by Image J software (Scion Corp., Frederick, MD).

### Cell invasion assay (transwell assay)

Transwell assay was performed to assess cell invasion capability. The 24-well BioCoat cell culture inserts (BD Biosciences, Bedford, MA, USA) with a polyethylene terephthalate membrane (8-μm porosity) were used, in which the membranes of upper chamber were coated with Matrigel (1mg/ml, BD) and then incubated for 6h at 37 °C. LNCaP/R or PC-3/R cells were treated for 24 h with vehicle control, 10 μM Quer, 5 nM Doc or 10 μM Quer + 5 nM Doc. Then about 1×10^5^ cells were added into the upper chamber in serum-free media, and 0.6 ml complete culture medium was added into the lower chamber. After incubation for 24h, the cells were washed twice with PBS, the remaining cells were wiped away using cotton swabs. Paraformaldehyde was used as fixation solution, and 0.1 % crystal violet were used to stain. The number of the invasion cells on the bottom surface of the membrane was recorded by imaging device and counted under a light microscope.

### Apoptosis analysis by flow cytometry

PC-3/R and LNCaP/R cells were added into 12 well plates and incubated for 24h, then exposed separately to one of the treatments, namely vehicle control, 20 μM Quer, 5 nM Doc or 20μM Quer + 5 nM Doc, in a humidified incubator for 24h at 37 °C. The cells were then washed with PBS and detached with 0.25% Trypsin-EDTA solution, before proceeding to resuspension in 100 mL of 1 X binding buffer. The cells were harvested and stained with Annexin V-PE and 7AAD according to the manufacturer's protocol. A total of 10,000 cells per sample were tested, then apoptotic cells were analyzed.

### Western blot analysis

To explore the mechanism underlying the effect of docetaxel and quercetin combination treatment on prostate cancer cells, LNCaP/R and PC-3/R cells were treated for 48h with one of the following reagents: vehicle control, 20 μM Quer, 5 nM Doc or 20 μM Quer + 5 nM Doc. Then in order to compare the differences among parental prostate cancer cells, docetaxel-resistant subclones and quercetin-treated resistant subclones, the groups of quercetin -treated resistant subclones were treated with 20 μM Quer for 48h, the other two groups were treated with vehicle control for 48h. Protein lysates were harvested on ice by scraping and mixing by vortex with ice-cold RIPA buffer (Beyotime Institute of Biotechnology, Beijing, China) containing protease inhibitor cocktail (Roche, Switzerland). The protein concentrations were quantified with the Bradford Protein Assay (Bio-Rad Laboratories, USA). After denatured at 100°C for 10 min, the protein samples were loaded and separated by SDS-PAGE and transferred onto a PVDF membrane. Nonspecific binding sites were blocked with 5% (w/v) fetal bovine serum at room temperature for 2h. Then, the membrane was incubated with mouse monoclonal antibody at 4°C overnight. The primary antibodies for testing were listed as follows: anti-β-actin, anti-PI3K, anti-p-Akt, anti-Akt, anti-Bax, anti-bcl-2, anti-AR, anti-PSA, anti-EpCAM, anti-Twist2, anti-E-cadherin and anti-P-gp antibody(at 1:1000 dilution respectively), and all antibodies were purchased from Abcam (Cambridge, MA, USA). After being washed with TBST buffer for 15 minutes three times, the membranes were incubated with HRP-labelled goat antimouse IgGs (1:2000; Abcam) at room temperature for 1h. Then, protein expression was detected by ECL and photographed by BioSpectrum Gel Imaging System (UVP, Upland, CA). β-actin was used as the loading control for Western blots.

### Animal study

All procedures carried out on mice were approved by the Experimental Animal Ethics Committee of Capital Medical University. For this part of the study, Male BALB/c nude mice 6 weeks old were used, which were obtained from the ministry of experimental animals of Capital Medical University. Mice were reared under specific pathogen-free environmental conditions, including 12h light-dark cycle at 23 ± 2°C and 58 ± 8% humidity. Animals had free access to food and drink. All mice adapted to the new surroundings for one week prior to the experiment. Quercetin was dissolved in 25% hydroxypropyl-β-cyclodextrin (HPβCD, w/v in ddH^2^O). Docetaxel solution was prepared by diluting stock solution (22.5 mg/mL in 100% EtOH) in diluent (1 L polysorbate in 18 L 5% glucose in distilled water) to a final concentration of 1.125 mg/mL. Vehicle solutions only contained corresponding solvent. 5×10^5^ PC-3 cells were suspended in 100 μL PBS, or 2×10^8^ LNCaP cells were suspended in 100 μL of matrigel, while PBS mixture (1:1) were injected subcutaneously into the right axillary fossa of mice. When the volume of xenograft tumors reached about 100 mm^3^, the intervention treatment started. Mice were randomly assigned to one of the four groups (n = 8 per group). Based on our *in vitro* results, preliminary experiments and many other researchers' studies 13, 18, the therapeutic schedule was set up as follows: (1) vehicle control group: injecting vehicle control of quercetin intraperitoneally every other day + vehicle control of docetaxel intravenously every 7 days; (2) docetaxel treated group: injecting vehicle control of quercetin intraperitoneally every other day + 10 mg/kg docetaxel intravenously every 7 days; (3) quercetin treated group: injecting 75 mg/kg quercetin intraperitoneally every other day + vehicle control of docetaxel intravenously every 7 days; (4) injecting 10 mg/kg docetaxel intravenously every 7 days + 75mg/kg quercetin intraperitoneally every other day. Tumor sizes were measured every 3 days using caliper, and tumor volumes were calculated according to the formula: L×S^2^×0.5, in which L represented the longest diameter and S represented the shortest diameter of tumor 19. These mice were anesthetized with chloral hydrate and sacrificed by cervical dislocation, and tumor tissues were weighted after 28 days. The tumor samples were collected to prepare the follow-up experiments.

### Immunohistochemistry (IHC) stain

At the end of each animal study, xenograft tumors were fixed in 10% phosphate buffered formalin and paraffin-embedded for immunohistochemical detection. 5 µm-thick sections were deparaffinized with xylene, rehydrated in an alcohol gradient, immersed in 3% H^2^O^2^, and then incubated with primary antibody Ki67 (1:1000, Abcam) at 4°C overnight. The processed sections were incubated with a secondary antibody using the ABC kit (Vector Laboratories. Burlingame, CA, USA) for 1h at room temperature. The resultant signals were visualized by diaminobenzidine reaction and counterstaining with hematoxylin. The number of Ki67 positive cells was analyzed from 3 random high-power fields of each slide. Sections with the absence of primary antibody and the same concentration of secondary antibody served as negative control.

### Statistical analysis

All statistical analysis was performed using SPSS (22.0), and the data were showed as mean ± standard deviation (SD). Statistical comparison among groups was performed as one-way analysis of variance (ANOVA), followed by Fisher's least significant differences (LSD) test. It was set that P value less than 0.05 represented statistical significance.

## Results

### Establishment of docetaxel-resistant prostate cancer cell lines

**Figure 1:**
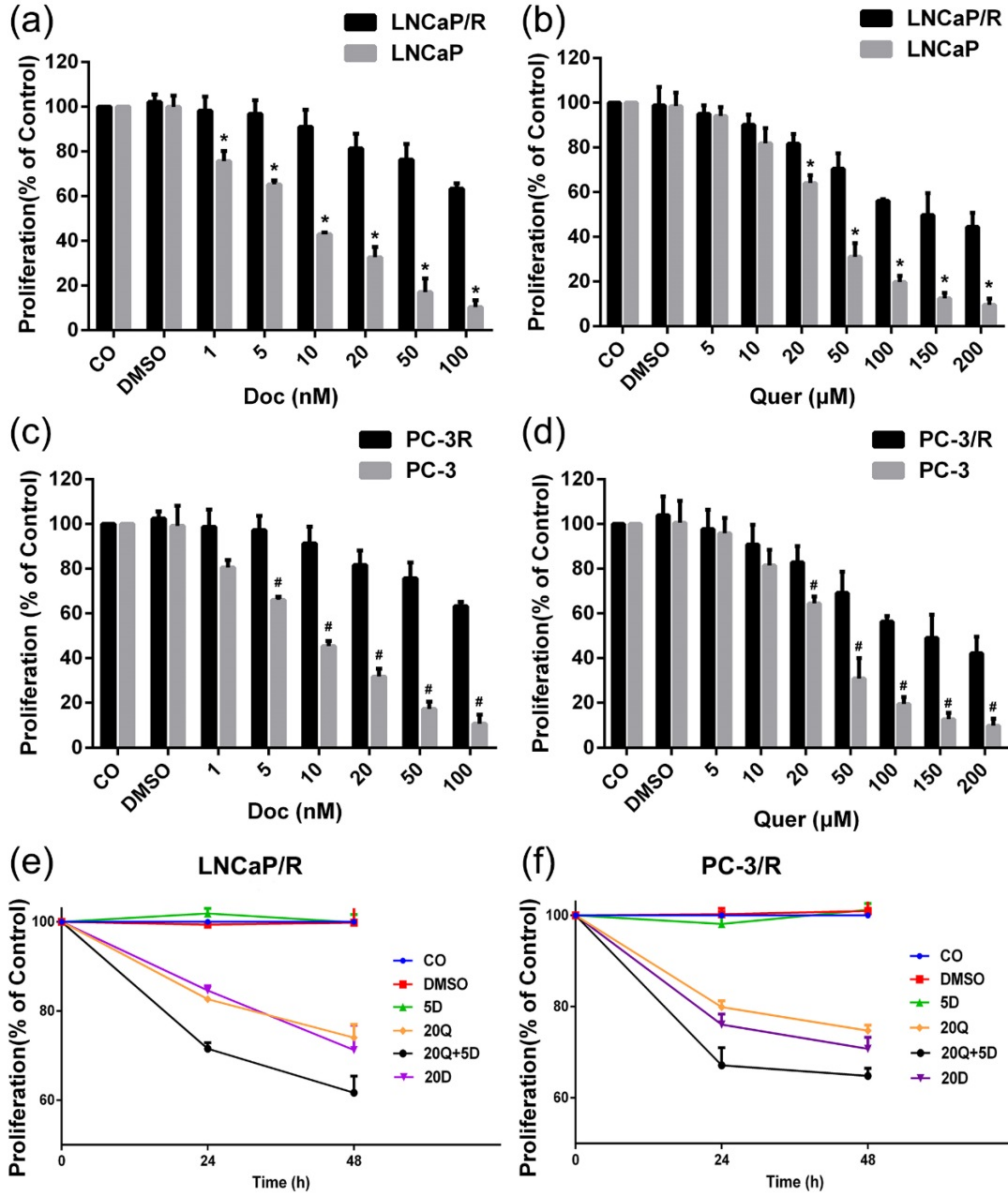
** Establishment of docetaxel-resistant prostate cancer cell lines and docetaxel resistant reversal effect of quercetin on proliferation.** The cells were treated with different concentrations of docetaxel or quercetin as indicated. 24h later, the viability was measured by CCK-8 assay: (a) LNCaP/R or LNCaP treated with docetaxel (nM); (b) LNCaP/R or LNCaP treated with quercetin (μM); (c) PC-3/R or PC-3 treated with docetaxel; (d) PC-3/R or PC-3 treated with quercetin. Likewise, LNCaP/R (e) or PC-3/R (f) were treated with different concentrations of docetaxel or quercetin or their combination therapy as indicated. Cell viability was determined by CCK-8 assay in 24h or 48h. Three independent experiments were performed at least in triplicates and the data are presented as means ± S.D. *P < 0.05, Compared with control group of LNCaP/R. #P < 0.05, Compared with control group of PC-3/R group.

In order to validate the docetaxel-resistant prostate cancer cell lines and establish the optimal experimental concentrations of docetaxel and quercetin for subsequent sections, the effects of 24h treatment with different concentrations of docetaxel or quercetin were compared by CCK-8 proliferation assay. After treatment with varied doses of docetaxel or quercetin for 24h, the proliferation of parental prostate cancer cells was inhibited in a dose-dependent manner (Figure 1a, 1b, 1c, 1d). Significant growth inhibition at low concentration of docetaxel (5 nM) was observed on docetaxel naïve cells. In contrast, at the same concentrations of docetaxel (5 nM), the cell proliferation was not affected in the docetaxel-resistant cell lines (Figure 1a & Figure 1c). In this way, docetaxel-resistant prostate cancer cell lines were validated. The DMSO solvent alone, at a dilution of 1: 1000, which was the lowest concentration of dilution of docetaxel or quercetin stock in medium, did not affect the cell viability, as expected.

### Docetaxel-resistance reversal effect of quercetin on cell proliferation, migration, invasion and colony formation

**Figure 2:**
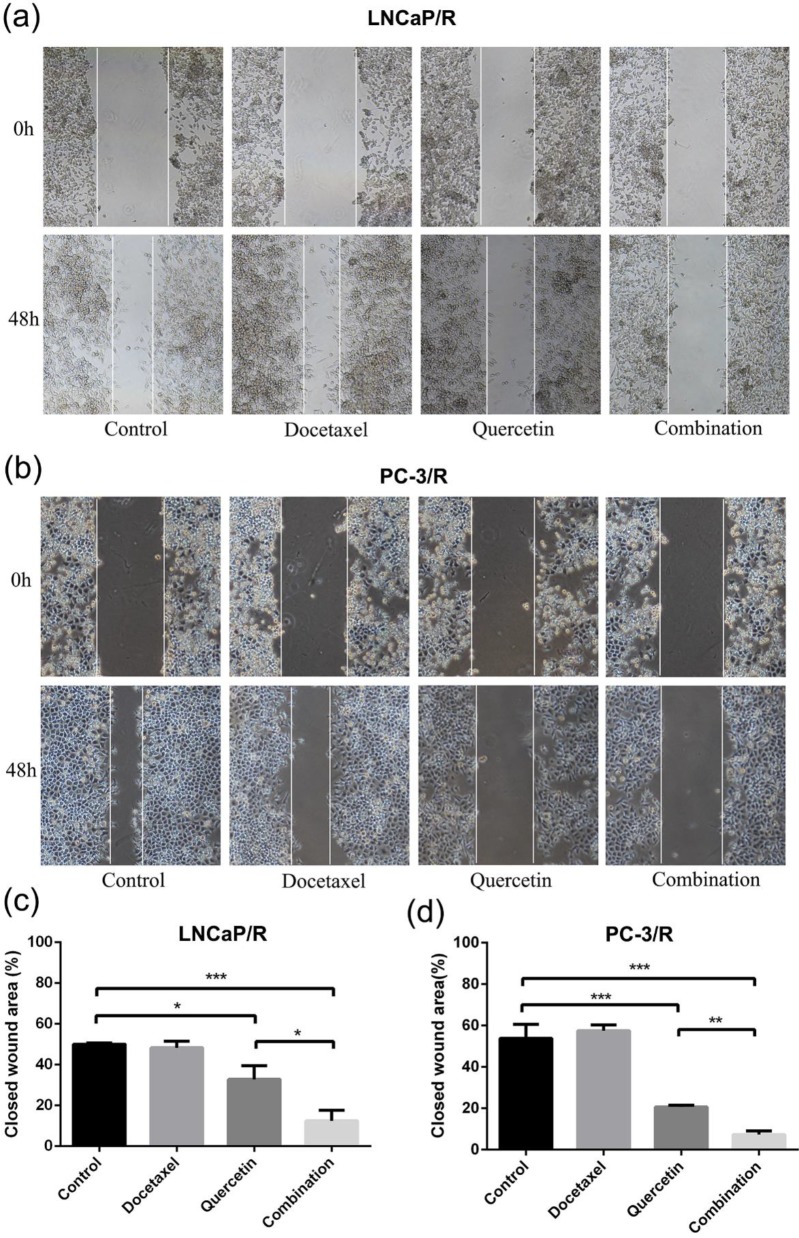
** Docetaxel resistant reversal effect of quercetin on migration.** The indicated treatment were assessed with respect to their effects on the migration in LNCaP/R cells (a, c) or PC-3/R cells (b, d). Three independent experiments were performed at least in triplicates and the data are presented as means ± S.D. *P < 0.05, **P < 0.01, ***P < 0.001.

**Figure 3:**
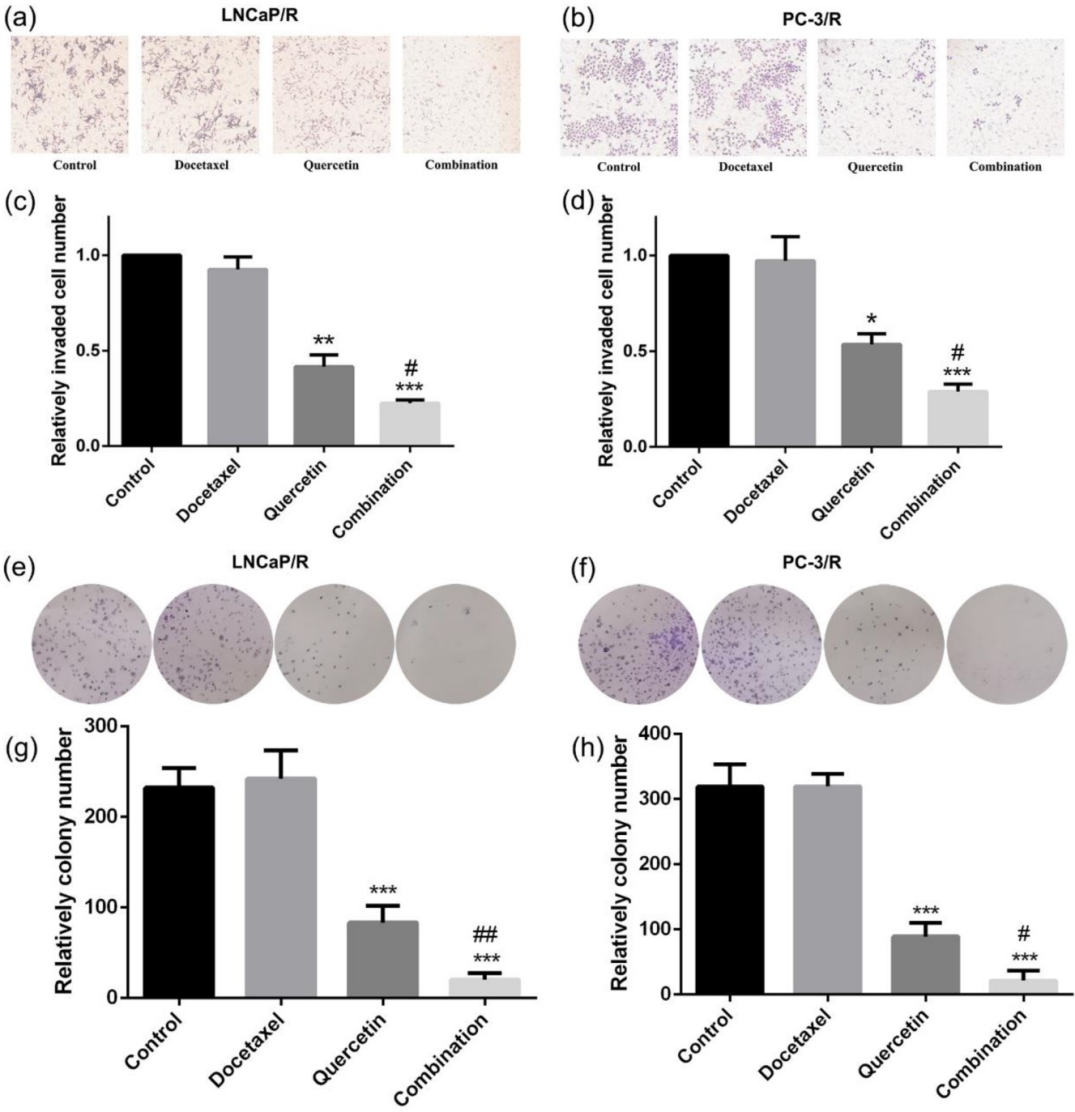
** Docetaxel resistant reversal effect of quercetin on invasion and colony formation.** The indicated treatment were assessed with respect to their effects on the invasion (a, b, c, d) and colony formation (e, f, g, h). Three independent experiments were performed at least in triplicates and the data are presented as means ± S.D. *P < 0.05, **P < 0.01, ***P < 0.001. Compared with vehicle control group. ^#^P < 0.05. Compared with quercetin group.

To research the effect of quercetin and docetaxel on biological function of docetaxel-resistant prostate cancer cells, cell proliferation assay (CCK-8), cell migration assay (wound healing assay), cell invasion assay (transwell assay) and colony formation assay were applied. Depending on the results of CCK-8 cell proliferation assay, Quer (20 μM) groups showed significant reduction in cell survival rate (CSR) not only at 24h but also at 48h. However, Doc alone at concentration of 5 nM failed to induce any anti-proliferative effect on LNCaP/R or PC-3/R cells. The cell survival rate of combination therapy group (20 μM Quer + 5nM Doc) was much lower than that of Quer (20 μM) only group (Figure 1e & 1f). To elucidate the migration capacity, wound healing assay was performed. The difference between LNCaP/R and PC-3/R cells was not significant. Although the wounds were slightly open in control groups and docetaxel groups, the difference between the two groups was insignificant (P >0.05, Figure 2). However, it was widely open in quercetin group, and moreover, the wound of combination therapy group was nearly not healing. To further define invasion potential, cell invasion transwell assay was performed. Likewise, Doc (5nM) alone did not inhibit the cell invasion compared to vehicle control (P >0.05, Figure 3). In LNCaP/R cells, the invasion potential through matrigel was significantly inhibited by Quer (20μM) compared to vehicle control. And the invasion potential was maximumly inhibited in combination group among the four groups. The results of PC-3/R cells were similar to those of LNCaP/R. Then, the cells were treated, and 48h later an equal number of surviving cells were seeded for colony formation. With the treatment of 5 nM Doc, the colony formation ability of LNCaP/R or PC-3/R cells was not inhibited at any significant level, compared with that of control group (P >0.05). Furthermore, the colony number of combination therapy group (20 μM Quer + 5 nM Doc) was much less than that of quercetin (20μM) only group (P <0.05). Taken together, these results indicated that quercetin had the reversal effect of docetaxel-resistance, which could inhibit cell proliferation, migration, invasion and colony formation of docetaxel-resistant prostate cancer cells.

### Docetaxel-resistance reversal effect of quercetin on apoptosis

**Figure 4:**
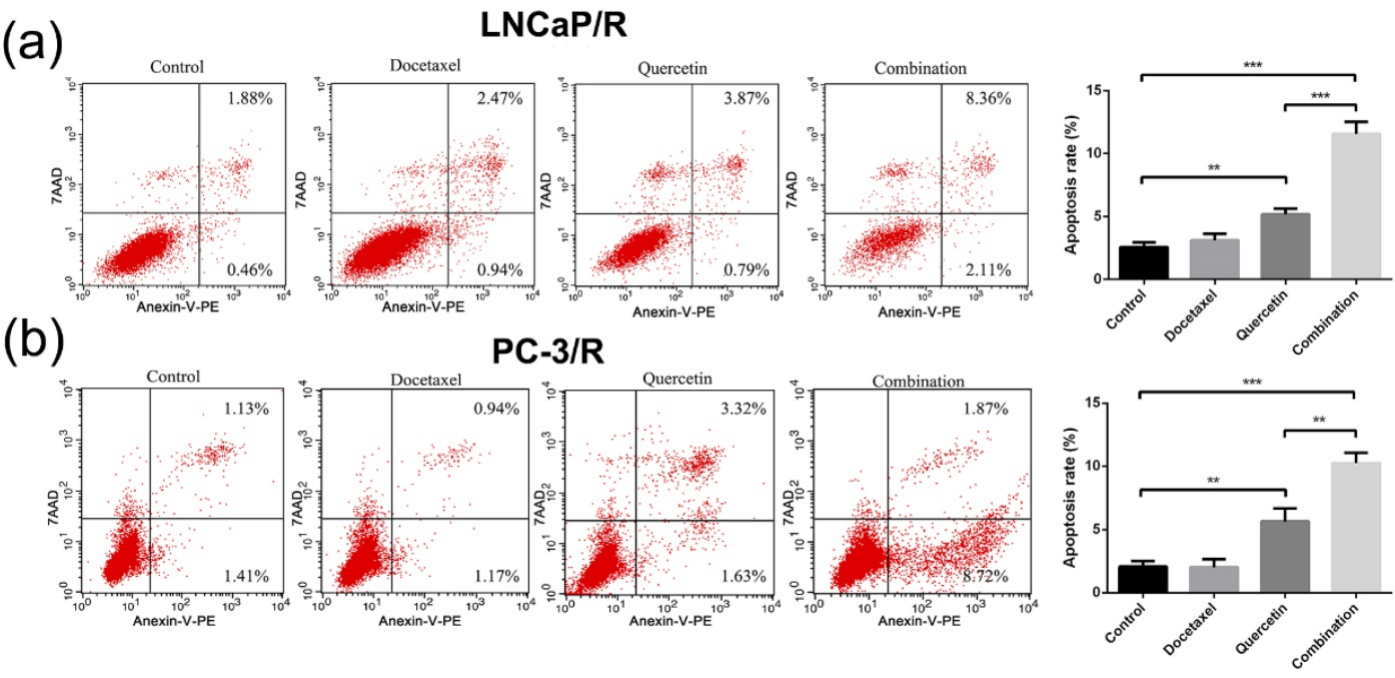
** Docetaxel resistant reversal effect of quercetin on apoptosis.** The dot plots (a) and the percentage (%) of cell distribution (c) of LNCaP/R cells and the dot plots (b) and the percentage (%) of cell distribution (d) of PC-3/R cells after 24 h treatment with indicated drugs or vehicle control as determined by Annexin V-7AAD and PE staining. Data are presented as mean ± SD. *P < 0.05; **P < 0.01; ***P < 0.001. These experiments were repeated at least three times.

The cell apoptosis of LNCaP/R and PC-3/R cells was subsequently investigated by flow cytometry. As shown in Figure 4, there was no valid apoptosis-inducing effect in the Doc (5nM) alone groups (Doc group vs control group: P >0.05). Additionally, the percentage of LNCaP/R and PC-3/R cells with positive Annexin V-positive staining (total apoptotic cells) was increased with the treatment of 20 μM Quer (Figure 4). However, the significant increase in apoptosis phase was observed after the treatment of combination therapy (20 μM Quer + 5nM Doc), compared with quercetin (20μM) only group (P <0.001). Overall, quercetin achieved its reversal effect of docetaxel resistance by inducing apoptosis activation for docetaxel-resistant prostate cancer cells.

### Docetaxel-resistance reversal effect of quercetin *in vivo*

**Figure 5:**
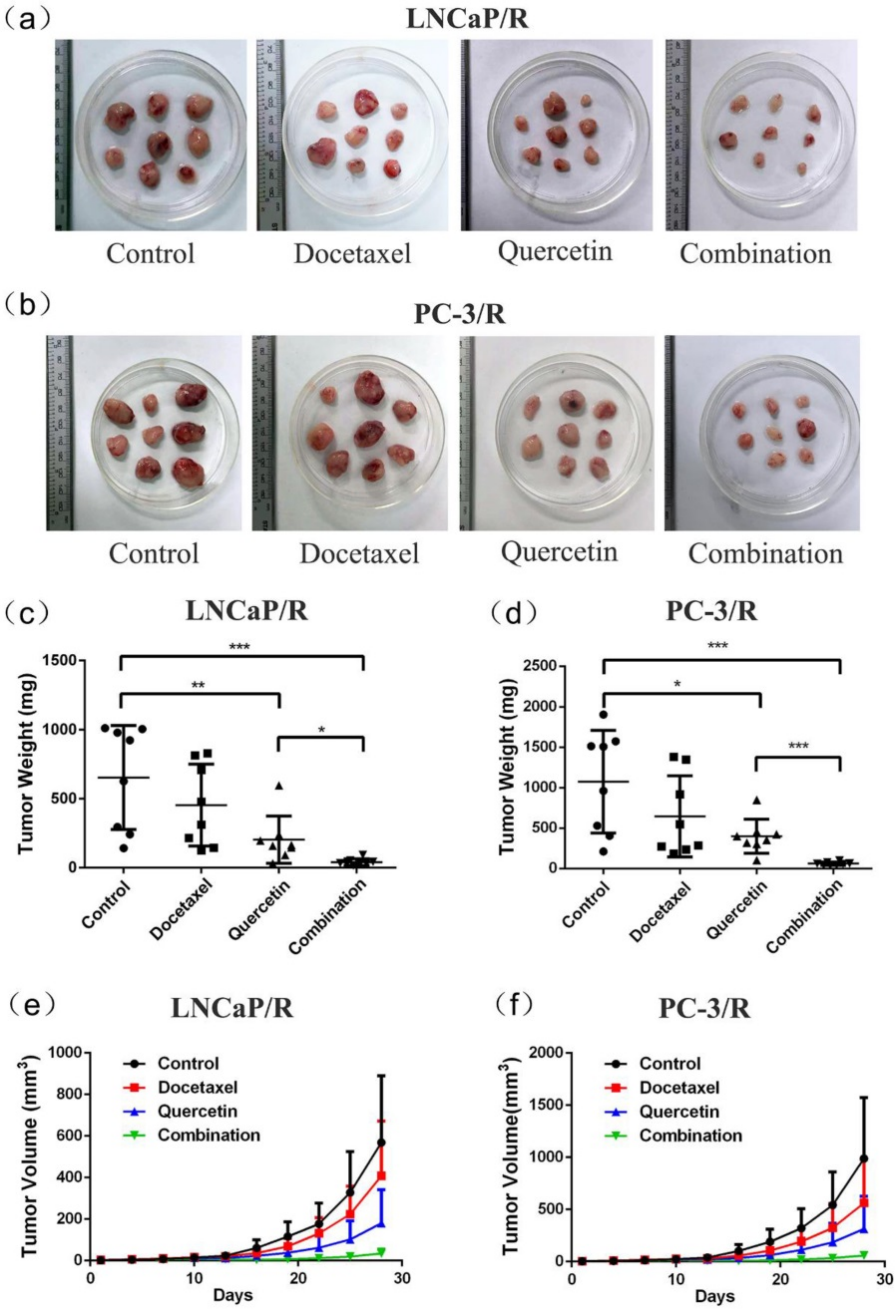
** Docetaxel resistant reversal effect of quercetin *in vivo*.** The indicated treatment were assessed as for their parts in modulating tumor size (a, b, e, f) and weight (c, d) of mice models, Data are presented as mean ± SD. *P < 0.05; **P < 0.01; ***P < 0.001.

**Figure 6:**
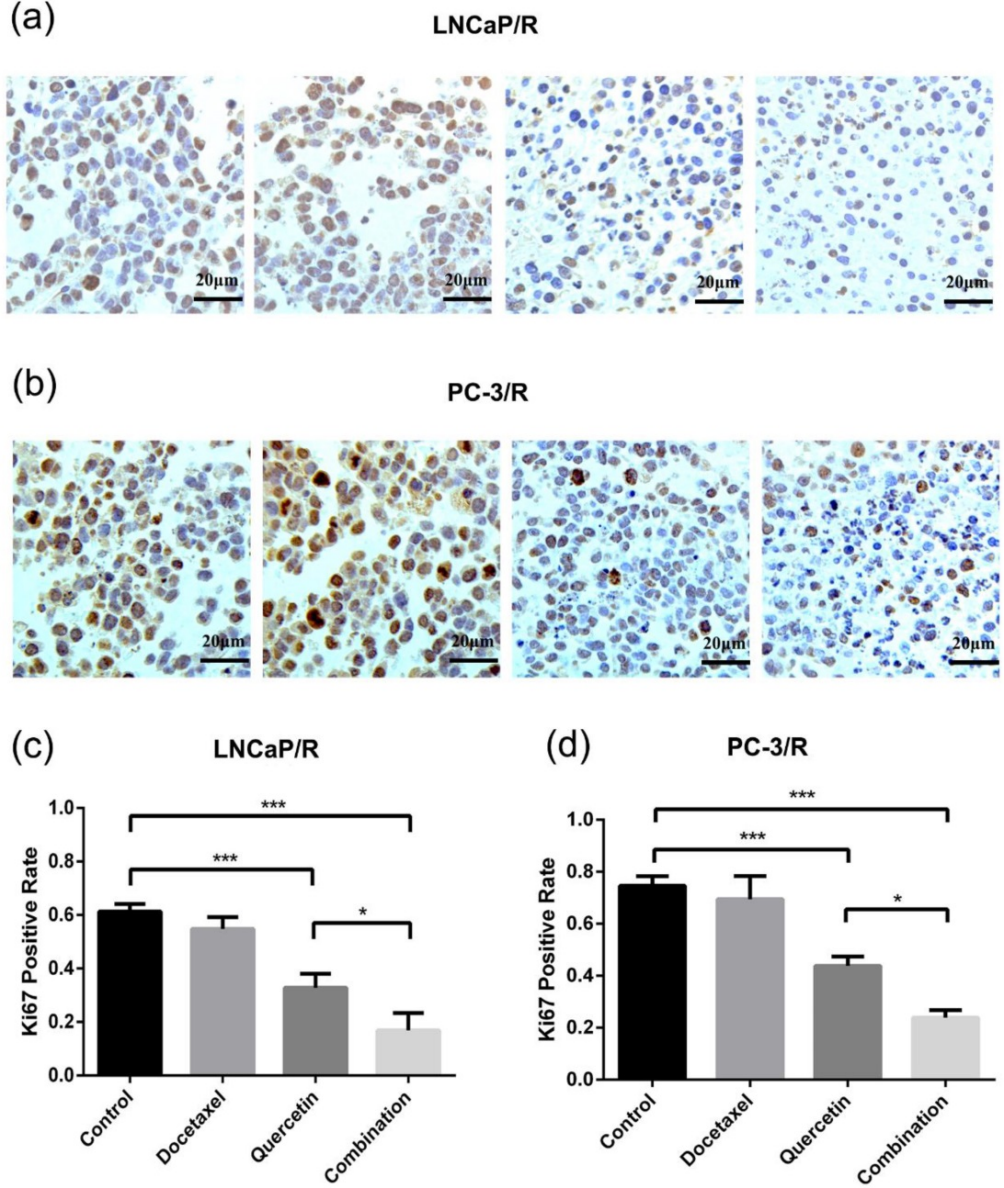
** Docetaxel resistant reversal effect of quercetin on inhibiting Ki67 expression in xenograft tumor tissues.** (a, b) Immunohistochemical detection showed Ki67 positive cells in LNCaP/R and PC-3/R xenograft tumor. (c, d). The number of Ki67 positive cells were represented as means ± SD, number was from three random high powered fields per slide (light microscopy, hpf, 400×). *P<0.05; ***P < 0.001.

To further validate the docetaxel-resistance reversal effect of quercetin *in vivo*, LNCaP/R and PC-3/R cells were injected to the nude mice to build *in-vivo* models. At first, these mice with LNCaP/R and PC-3/R xenograft tumors were treated with specified therapy, and the procedure lasted for 28 days, during which tumor sizes were recorded every three days. The tumor size of docetaxel groups increased as rapidly as that of the control groups (Figure 5e & 5f). However, the tumor size of quercetin groups increased more slowly than those of the above two groups. Compared with the tumor growth in quercetin group, the tumors in combination group grew even more slowly. At 28 days, the tumors were isolated and weighed (5a & 5b). The tumor weights of docetaxel groups and control groups were the heaviest (Figure 5c & 5d), while the tumor weight of quercetin only group was lighter than tumor weight of the above two groups. Tumor weight of combination therapy group was the lightest. In addition, immunohistochemistry analysis on Ki67 expression, the index of cell proliferation (Figure 6a & 6b), showed the inhibitory effect of docetaxel or quercetin on tumor cell proliferation *in vivo*. Docetaxel alone could not reduce Ki67 expression (Doc group vs Control group: P >0.05, Figure 6c & 6d), while quercetin reduced Ki67 expression significantly (Control group vs Quer group: p<0.001, Figure 6c & 6d). And combination therapy showed the strongest inhibitory effect on tumor cell proliferation. Taken together, these results implied that quercetin had docetaxel-resistance reversal effect *in vivo*.

### Effect of docetaxel and quercetin combination treatment on apoptotic protein expression and PI3K/Akt pathway

**Figure 7:**
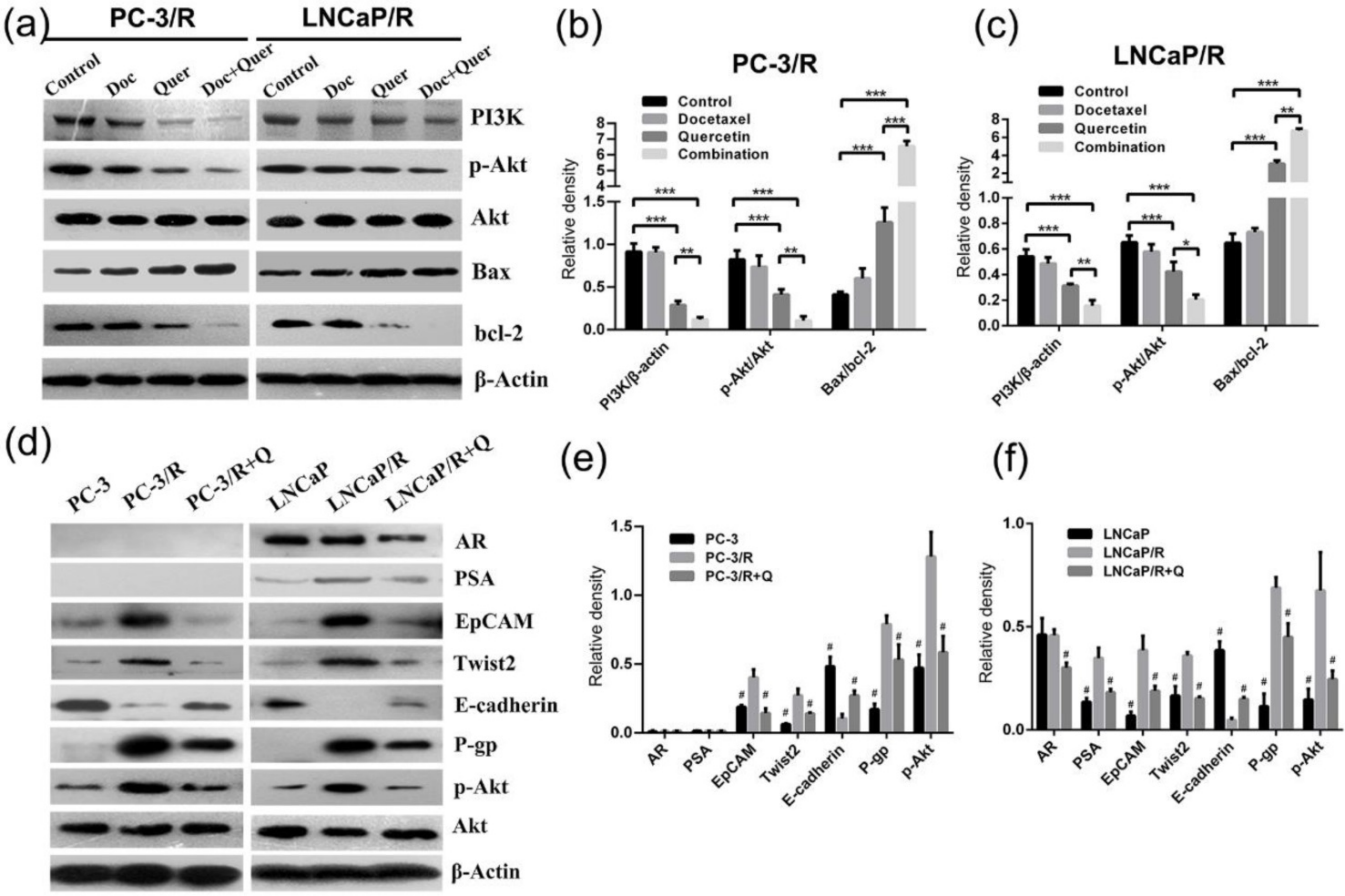
**The indicated treatment were assessed regarding their role in altering the expression of proteins.** (a). PI3K, pAkt, Akt, Bax, bcl-2 protein expression of indicated groups were examined by western blot. Relative density is the ratio of two indicated proteins (b, c). Quercetin reverses docetaxel resistance by reversing the up-regulation of P-gp, EMT, stem-like cell phenotypes and the activation of androgen receptor and PI3K/Akt signal pathways (d). Relative density is the ratio of indicated protein to β-actin (e, f), which were represented as means ± SD (values from three independent experiments). (b, c) *P < 0.05; **P < 0.01; ***P < 0.001; (e, f) #P < 0.05. Compared with PC-3/R group or LNCaP/R group.

To explore the mechanism underlying the effect of docetaxel and quercetin combination treatment on prostate cancer, the apoptosis protein (Bax and Bcl-2) expression level and the level of activity via a classic signaling pathway (PI3K/Akt pathway) was evaluated by Western blotting (Figure 7a). Western blot showed that docetaxel alone could not apply any effect on the expression of apoptosis proteins in LNCaP/R and PC-3/R cells (P >0.05, Figure 7b & 7c), while quercetin induced apoptosis by regulating the expression proapoptotic protein Bax and anti-apoptotic protein Bcl-2, and the combined therapy had the strongest effect. The apoptosis-inducing effect was determined by the ratio of Bax/Bcl-2 (Figure 7b & 7c). While it was generally recognized that PI3K/Akt pathway played a pivotal role in docetaxel chemo-resistance 20-22.Western blot showed that the phosphorylation of Akt and the expression of PI3K were suppressed most significantly depending on the combination treatment of docetaxel and quercetin.

### Quercetin reverses docetaxel resistance by reversing the up-regulation of P-gp, the mesenchymal and stem-like cell phenotypes, the activation of androgen receptor and PI3K/Akt signaling pathways

P-gp played a direct role in the resistance to many chemotherapeutics 23, 24. In order to investigate whether efflux ATP-binding cassette (ABC) transporter proteins were involved in the reversal of docetaxel and quercetin, the expression of P-gp was analyzed by Western blotting (Figure 7d), which showed that the expressions of P-gp in LNCaP/R and PC-3/R cells were significantly higher than that of their parental prostate cancer cells (p<0.05, Figure 7e & 7f). However, after treatment of quercetin, the expression of P-gp in LNCaP/R and PC-3/R cells was significantly reduced. Moreover, it was reported that the mesenchymal and stem-like cell phenotypes were strongly associated with docetaxel resistance 25. Therefore, the expression levels of the stemness markers EpCAM, the mesenchymal cell marker Twist2 and the epithelial cell marker E-cadherin were evaluated by western blot (Figure 7d). These proteins were also considered to be prognostic markers of chemotherapy. EpCAM and Twist2 were up-regulated in LNCaP/R and PC-3/R cells, E-cadherin was down-regulated, compared with the parental LNCaP and PC-3 cells (P <0.05, Figure 7e & 7f). Whereas the treatment with quercetin decreased the expression of EpCAM, Twist2 and increased that of E-cadherin. Next, two important androgen signal pathway markers of prostate cancer were tested by Western blot, including androgen receptor (AR) and PSA 26. It was found that PC-3 and its docetaxel-resistant subclone PC-3/R failed to express AR or PSA. And in LNCaP/R cells, there was no significant difference in AR expression between LNCaP group and LNCaP/R group (P >0.05, Figure 7e & 7f), while AR expression was reduced in quercetin treated LNCaP/R group (P <0.05). Moreover, the expression of PSA in LNCaP/R was increased compared to the parental LNCaP, while the expression of PSA was significantly decreased after treated by quercetin (P <0.05). Then, Akt and p-Akt protein expression profiles were investigated by Western blot. There was no significant difference in Akt protein expression among groups (P >0.05). Whereas p-Akt expression was up-regulated in LNCaP/R and PC-3/R cells as compared to parental LNCaP and PC-3 cells. After treated with quercetin, the expression of p-Akt was remarkably reduced in LNCaP/R and PC-3/R cells (P <0.05).

## Discussion

Docetaxel‑based therapy is the standard first‑line chemotherapy in patients with metastatic prostate cancer. Although it has gained great success in the inhibition or controlling the growth and metastasis of prostate cancer, the emergence of chemoresistance and dose-dependent adverse reactions limits its efficacy. Quercetin can apply its promising anti-cancer property in prostate cancer by targeting various signal pathways 12, 27. Moreover, quercetin was reported as an efficient agent to reverse chemo-resistances in hepatocellular carcinoma and breast cancer 28-30. Studies by Wang et al. showed that combining green tea and quercetin could sensitize CRPC to docetaxel 17. Based on the previous studies, it was supposed that quercetin could reverse docetaxel resistance in prostate cancer. In the present study, two docetaxel-resistant prostate cancer cell lines (LNCaP/R and PC-3/R) were developed from PC-3 and LNCaP cells by exposing cells to gradually increasing concentrations of docetaxel. The resistance to docetaxel of the subclones were validated by proliferation assay. At a certain concentration, although the administration of docetaxel alone had little effect on resistant cells, the treatment of quercetin still worked. More importantly, the combination therapy of docetaxel quercetin caused much more improved than quercetin alone. The combined therapy could considerably inhibit cell migration, invasion, colony formation and reduce cell viability. It was also associated with increased apoptosis and inhibition of PI3K/Akt signal pathway. *In-vivo* study showed that combined use of quercetin and docetaxel could inhibit docetaxel-resistant xenograft tumor growth to the largest extent and lead to the least Ki67 expression. These results suggested that resistant cells could become sensitive to docetaxel again after being treated by quercetin. In the CCK-8 proliferation assay (Figure 1e & f), 20 μM Quer + 5 nM Doc could achieved improved efficacy compared 20 nM Doc alone. So, it was suggested to add quercetin to the original chemotherapy regimen rather than increasing docetaxel dosage. Furthermore, combining quercetin with docetaxel might be more cost-effective and cause fewer side effects than docetaxel alone, which required further research works to verify.

**Figure 8:**
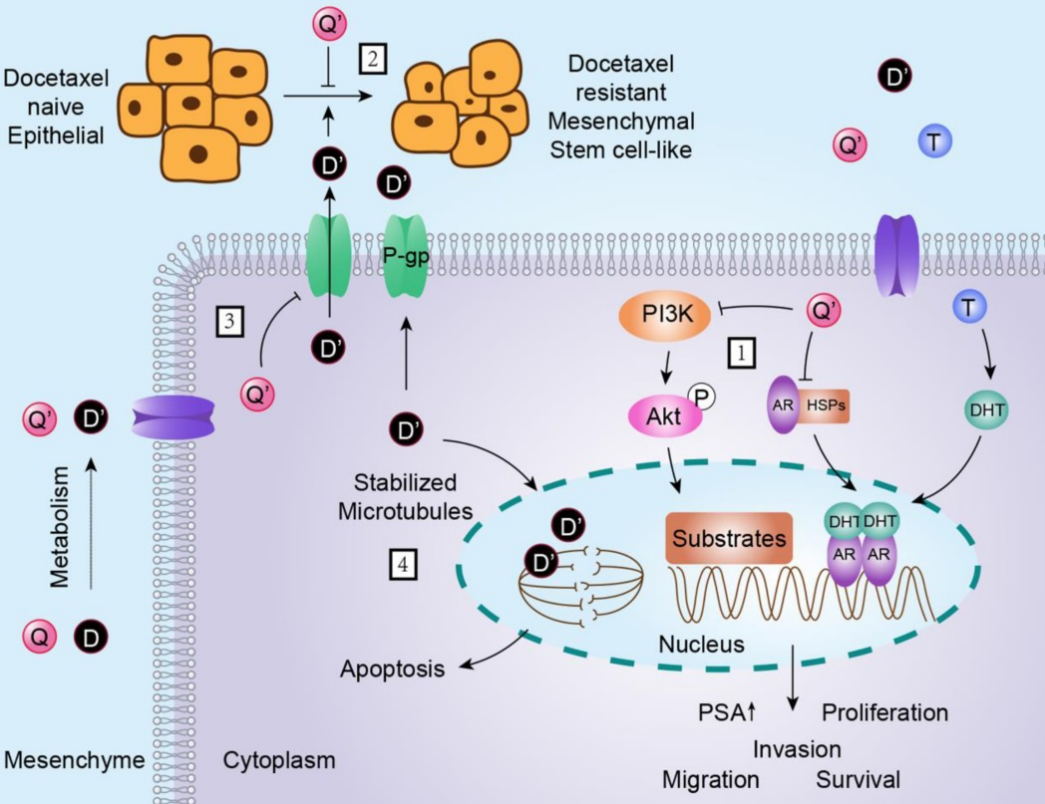
** Schematic diagram showing the role of quercetin in reversal effect of docetaxel resistance.** As depicted: (1) quercetin inhibits androgen receptor and PI3K/Akt signal pathway; (2) quercetin reverses EMT (epithelial-mesenchymal transition) and stem-like cell phenotypes of docetaxel-resistant prostate cancer cells; (3) docetaxel up-regulates P-gp, quercetin down-regulates P-gp; (4) although docetaxel can induce apoptosis through stabilizing microtubule, P-gp pumps out docetaxel to keep it at a low level in cytoplasm. Q is short for quercetin; Q' is short for quercetin metabolites; D is short for docetaxel; D' is short for docetaxel metabolites, P is short for phosphorylation; T is short for testosterone; DHT is short for double hydrogen testosterone; AR is short for androgen receptor; HSPs is short for heat shock proteins. The → indicates activation or induction, and → indicates inhibition or blockade.

The mechanism underlying docetaxel resistance was complex. In order to investigate which mechanisms could contribute to the reversal effect, we investigated the differences among parental prostate cancer cells, docetaxel-resistant subclones and quercetin treated docetaxel-resistant subclones. The results demonstrated that quercetin reversed docetaxel resistance in prostate cancer via the mechanisms as follows (Figure 8): the activation of androgen receptor and PI3K/Akt pathway, the appearance of remarkable mesenchymal and stem-like cell phenotypes, and the P-gp expression.

As far as we knew, one major cause of resistance to docetaxel therapy was the abnormal activation of androgen receptor (AR) signaling pathway. These mechanisms included AR over-expression, AR gene amplification, mutations, alterations in coregulators, and continuous androgens release from the tumor tissue or adrenal glands. The activation of AR signaling pathway by androgens not only increased cell proliferation but also repressed the apoptosis of prostate cancer cells 26, 31, 32. PC-3 cells could not express AR or PSA congenitally. In the present study, we found that there was no significant difference in the expression of AR between LNCaP and LNCaP/R cells. However, PSA, which was an AR downstream protein, was up-regulated in LNCaP/R compared to that in LNCaP. Quercetin treated LNCaP/R cells expressed less AR and the downstream protein PSA than LNCaP/R groups, which indicated the potential of quercetin to reverse docetaxel resistance in an androgen-dependent manner.

EMT (epithelial-mesenchymal transition) and stem-like cell phenotypes have been proved to be strongly associated with docetaxel resistance. Marin et al. found that EMT played an important role in docetaxel resistance by regulating the expression of the transcription factor ZEB1, a key mediator of EMT. And they observed that ZEB1 genetic down-modulation restored CDH1 expression but suppressed CD44 expression, which was consistent with the reversion of EMT and stem-like cell features 25. In the present study, we found that the mesenchymal cell marker Twist2 and the stemness markers EpCAM were up-regulated in LNCaP/R and PC-3/R cells, while the epithelial cell marker E-cadherin was down-regulated. Whereas treatment with quercetin decreased the expression of Twist2 and EpCAM and increased that of E-cadherin. As a result, our results confirmed the mechanistic role of EMT and stem-like cell phenotypes in docetaxel resistance, and quercetin could reverse docetaxel resistance by reversing mesenchymal and stem-like cell phenotypes.

P-glycoprotein is an important factor of chemo-resistance, which acts as drug efflux pump for multiple chemotherapy drugs, including docetaxel 23. P-glycoprotein is encoded by the multidrug resistance-1 (MDR1) gene, which is over-expressed in docetaxel-resistant cancer cells 24. In the present study, P-glycoprotein expression of docetaxel-resistant prostate cancer cell lines was increased, which could be reversed by being treated with quercetin. Overall, our results strongly suggested that quercetin could reverse docetaxel resistance partly through decreasing P-glycoprotein expression.

Another major cause of resistance to docetaxel therapy is the abnormal activation of PI3K/Akt signaling pathway. The activation of PI3K/Akt signal pathway could lead to overexpressed multi-drug resistance proteins, as well as the upregulation of various oncogenes and growth factors such as VEGF, c-myc and cyclin D1 21, 22. Our results suggested that PI3K/Akt signaling pathway was excessively activated after prostate cancer cells developed resistance to docetaxel. And quercetin could also reverse the activation of this pathway. Based on the results, quercetin could reverse docetaxel-resistance at least partly through decreasing activation of PI3K/Akt signaling pathway.

## Conclusion

In the past, almost all studies on docetaxel therapy in prostate cancer only focused on CRPC. To comprehensively interpret the clinical significance, the present research demonstrated that quercetin had docetaxel-resistance reversal effect in prostate cancer both in HSPC and CRPC. As a result, the present study complied with the treatment concept at present and provided substantial support for the clinical trial of quercetin in docetaxel-resistant prostate cancer.

## Abbreviations

Doc: docetaxel
AR: androgen receptor
FOXO1: Forkhead box O1
Quer: quercetin
mCRPC: metastatic castration resistant prostate cancer
mHSPC: metastatic hormone sensitive prostate cancer
HSPC: hormone sensitive prostate cancer
CRPC: castration resistant prostate cancer
DMSO: dimethyl sulfoxide
OD: optical density
